# Shared decision making and advance care planning: a systematic literature review and novel decision-making model

**DOI:** 10.1186/s12910-023-00944-7

**Published:** 2023-08-14

**Authors:** Ana Rosca, Isabelle Karzig-Roduner, Jürgen Kasper, Niek Rogger, Daniel Drewniak, Tanja Krones

**Affiliations:** 1 Clinical ethics, Stadtspital Zürich, Birmensdorferstrasse 497, Zürich, 8063 Switzerland; 2 Institute of Biomedical Ethics and History of Medicine, Clinical Ethics, University of Zürich, University Hospital Zürich, Zürich, Switzerland; 3 Department of Nursing and Health Promotion, Oslo metropolitan Universita, Oslo, Norway; 4 University of Applied Sciences Leiden, Leiden, Netherlands; 5 Institute of Biomedical Ethics and History of Medicine, University of Zürich, Zürich, Switzerland

**Keywords:** Shared decision making, Advance care planning, Aortic stenosis, Systematic literature review, Integrative model

## Abstract

**Background and Aims:**

Shared decision making (SDM) and advance care planning (ACP) are important evidence and ethics based concepts that can be translated in communication tools to aid the treatment decision-making process. Although both have been recommended in the care of patients with risks of complications, they have not yet been described as two components of one single process. In this paper we aim to (1) assess how SDM and ACP is being applied, choosing patients with aortic stenosis with high and moderate treatment complication risks such as bleeding or stroke as an example, and (2) propose a model to best combine the two concepts and integrate them in the care process.

**Methods:**

In order to assess how SDM and ACP is applied in usual care, we have performed a systematic literature review. The included studies have been analysed by means of thematic analysis as well as abductive reasoning to determine which SDM and ACP steps are applied as well as to propose a model of combining the two concepts into one process.

**Results:**

The search in Medline, Cinahl, Embase, Scopus, Web of science, Psychinfo and Cochrane revealed 15 studies. Eleven describe various steps of SDM while four studies discuss the documentation of goals of care. Based on the review results and existing evidence we propose a model that combines SDM and ACP in one process for a complete patient informed choice.

**Conclusion:**

To be able to make informed choices about immediate and future care, patients should be engaged in both SDM and ACP decision-making processes. This allows for an iterative process in which each important decision-maker can share their expertise and concerns regarding the care planning and advance care planning. This would help to better structure and prioritize information while creating a trustful and respectful relationship between the participants. **PROSPERO 2019.** CRD42019124575

**Supplementary Information:**

The online version contains supplementary material available at 10.1186/s12910-023-00944-7.

## Background

Modern ethical codes recommend effective communication, trust and respect for ones’ dignity as some of many important requirements for a patient–clinician relationship [1–3]. As patients have distinct personalities, character, experiences, disease specific situations and cultural backgrounds [3], they also have different needs regarding how their autonomy is best supported.

According to Beauchamp and Childress, autonomous patients can choose and act intentionally, with understanding, and without controlling influences that determine their actions [4]. As individuals may not have the resources to make fully autonomous decisions in every situation, or may not want to decide on their own, the concept of relational autonomy was proposed. It emphasizes that autonomous choices are generally achieved or realized over time in the context of positive and negative social relations, and accepts various modes of patient engagement and empowerment within the decision-making process [5]. This approach stresses the importance of having collaborative dynamics in the relationship between patients and other key decision-makers. Shared decision-making (SDM) and advance care planning (ACP) are two evidence and ethically based concepts. They foster patient autonomy by engaging patients, their relatives and healthcare professionals (HCP) in the decision-making process. Both concepts are often described as best practice models and are included in medical curricula (e.g. CANMEDS [6]) and guidelines of medical professions (e.g. AHA [7]).

**Fig. 1:**
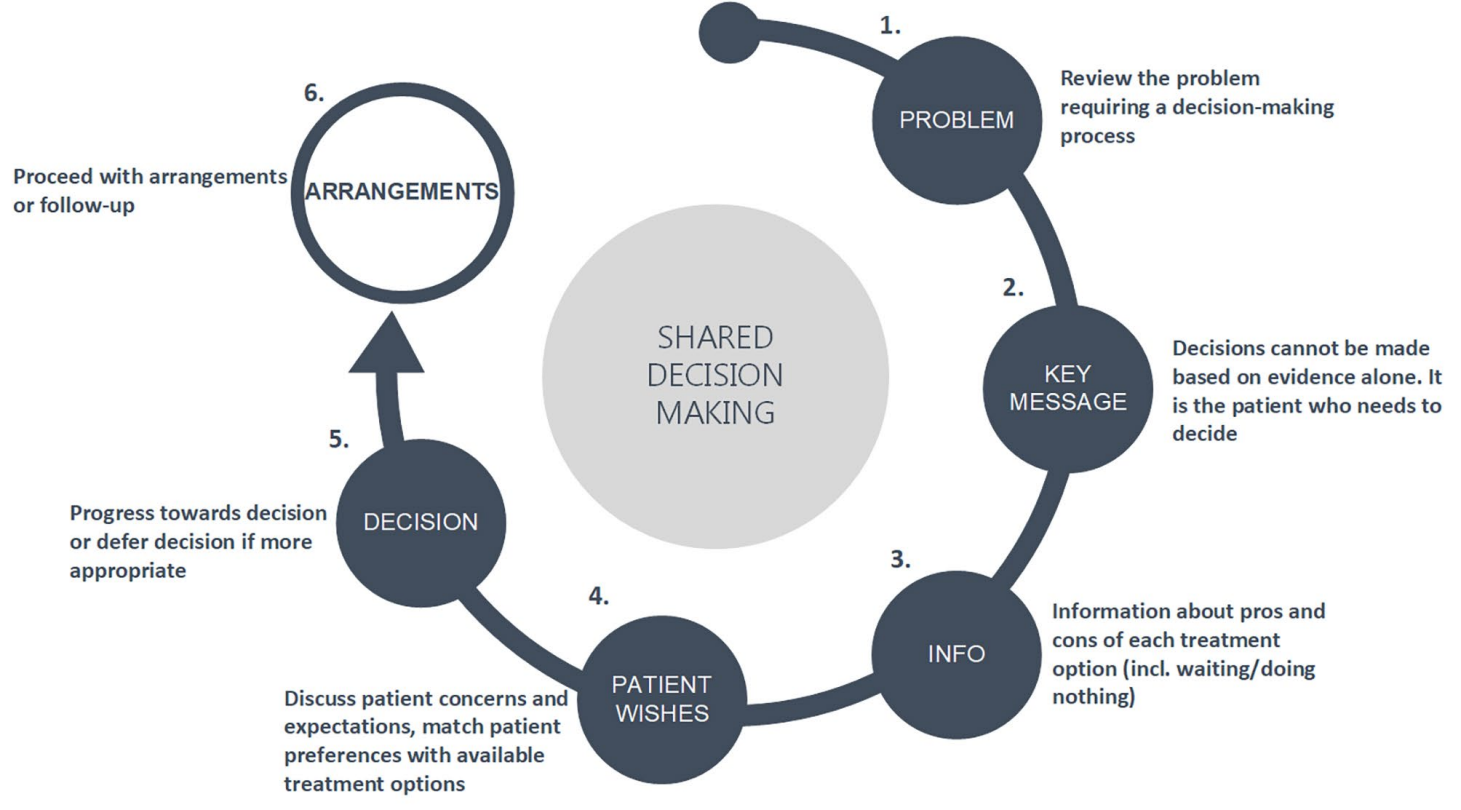
Shared decision making process as described by Makoul and Clayman [13]

SDM is a two-way information process between HCPs and patients, sharing medical and risk information as well as preferences and concerns to reach the best individual decision by negotiation [8, 9]. Although the SDM scientific community agrees on the overall structure of SDM, and efforts have been made to expand it to integrate goals of care deliberation [10] and significant others (family or friends) in the SDM process as an additional source of decision support and preference deliberation [11, 12], there is no internationally established SDM standard. Various reviews [13, 14] showed however that the SDM elements described in the literature can be summarized in six steps (Fig. 1). Probably one of the most important SDM steps is summarized as the “key message” [15] in Fig. 1, and it stresses out the importance of patient’s self-efficacy [13], their goals of care [10] or awareness of choice [14]. This step aims to explicitly convey that “decisions cannot be made based on evidence alone, it is the person who needs to decide”[15]. Respect for autonomy demands that a person should be appropriately informed that the evidence may be lacking, of poor quality or inconclusive, and the available treatment options may often manifest variations of benefits and risks for individual patients. Therefore, it is up to individuals to determine whether the benefits balance out the risks, and which uncertainties they are most willing to accept. Once the benefits and risks of all options have been discussed (Fig. 1 step 3) and the individual is not yet ready to express a preference for a treatment option, the HCP may either make a treatment recommendation or postpone the decision to a later point in time.

Regardless of the treatment choice, health complications like stroke or internal bleeding may arise during or shortly after an intervention, decided upon during an SDM process. These complications could limit patients’ decision-making capacity for subsequent treatment choices. It can alter the prognosis compared to the situation discussed during an SDM process (before the intervention), without the possibility of the patient to decide regarding the goals of care and treatment options of the arized complications. To respect their individual preferences during these deteriorating health conditions, patients should be enabled to anticipate and communicate their treatment wishes for such disease situations prior to the treatment decision. ACP is a concept developed to promote patient-centered care for situations in which the person lost the decision-making capacity and cannot speak for himself or herself. It is an iterative process involving patients, their surrogates and HCPs to discuss their goals of care and treatment options in case of temporary or permanent loss of decision-making capacity [16–18]. This may concern unforeseeable situations, such as an accident or sudden serious illness, as well as planned situations with incapacity for decision-making during interventions and operations with general anesthesia and in case of severe complications [19, 20]. In the ACP process, patients are empowered to communicate their goals of care with regard to 1) life prolonging treatment by all means, 2) life prolonging depending on prognosis/outcomes and/or 3) palliative/supportive care. ACP aims to collect patient’s values and treatment preferences in case of future incapacity of decision making in emergencies, prolongated or permanent loss of decision making capacity, and to document them in a written plan or an advance directive. These may include distinctly formulated preferences that are situation-specific and depend on the illness progression (if an illness is already present), or in case of unwanted outcomes after an unforeseen health crisis that may happen to any person. ACP can therefore influence the current treatment decisions, however, current treatment decisions may also trigger questions about ACP.

An ACP Delphi study differentiates between five important ACP elements: (1) care consistent with patient’s goals, (2) designation of a surrogate decision-maker, (3) documentation of the surrogate decision maker, (4) discussions with surrogate and (5) accesability of the documented and recorded patient wishes [21].

Both SDM and ACP are concepts, which also support patients with moderate or high risk of complications to express their autonomy in the decision-making process for immediate and future care. Within best practice literature, current medical curricula and existing guidelines, both concepts are cited and recommend for use in usual care. Although they share the common goal of fostering person centred care and well informed choices, there is no explicit literature that combines the two concepts into one single process, nor are there any documented examples on how the two concepts are being applied.

In this paper we aim to (1) assess how SDM and ACP is being applied using the care of patients with aortic stenosis (AS) as an example, and (2) propose a model to best combine the two tools and integrate it in the care process.

To reach these aims, we looked into how SDM and ACP are being used in the care of people with aortic stenosis (AS). It is a progressive fatal disease that occurs in 0.3–0.5% of the general population, and severe AS occurs in 3–4% in people older than 75 years of age, for which an invasive treatment is advised [22]. AS can be managed either by surgical aortic valve replacement (SAVR), transcatheter aortic valve implantation (TAVI), or symptoms can be managed with palliative care. As all treatment modalities come with specific benefits and risks (severe bleeding or ischemic stroke) with an elevated chance of being temporarly or even permanently unable to make autonomous decisions, current AS management guidelines recommend shared decision-making (SDM) [7] and advance care planning (ACP) [23, 24] to facilitate informed patient choices for these patients before a treatment decision. We therefore chose to conduct a systematic literature review on the use of SDM and ACP in the usual care of patients with AS. Based on this patient group we hope to be able to provide a model applicable for other cohorts in which both concepts are recommended as a standard for good care.

## Methods

### Systematic literature review

We conducted a systematic literature search in CINAHL, Cochrane, EMBASE, MEDLINE, PsychINFO, Scopus and Web of Science (see supplementary file 1 for search strategy). We included all empirical studies written in English or German, which focus on patients with AS, their surrogates and/or their healthcare professional (HCP). We searched for studies that described SDM and/or ACP or other communication interventions used to aid the decision-making process (see supplementary file 1 for inclusion criteria). The first search was performed on April 11th, 2019 and the repeated final search was performed on April 24th, 2023. Three researchers (AR, NR, DD) independently screened titles and abstracts against the above described inclusion criteria. We then assessed the full texts according to the inclusion criteria for a definitive study inclusion. Disagreements were solved by discourse and inclusion of a fourth researcher (AR, NR, DD and TK). Critical Appraisal Skills Program (CASP) was used to decide on the trustworthiness, relevance and results of all included papers [25]. The results of each included study have been individually screened, analyzed and synthesized using MAXQDA® software. By means of thematic analysis, we have critically and systematically analyzed and synthesized the findings with the purpose of determining the status quo and distinguish between the various processes leading to a treatment decision. This hermeneutic process is required to reach the second aim of our study: to suggest an integrative model for SDM and ACP for the peri interventional situation.

### SDM and ACP model

For the second purpose, the team (AR, IKR, JK and TK) built its data analysis process on the concept of empirical ethics. The process implies combining empirics (in this case the results provided by the review) with abductive reasoning (TK, JK) for the subsequent development of new understandings and concepts that may complement the ones more broadly used (AR, IKR, JK and TK) [26].

## Results

### Systematic literature review

**Fig. 2:**
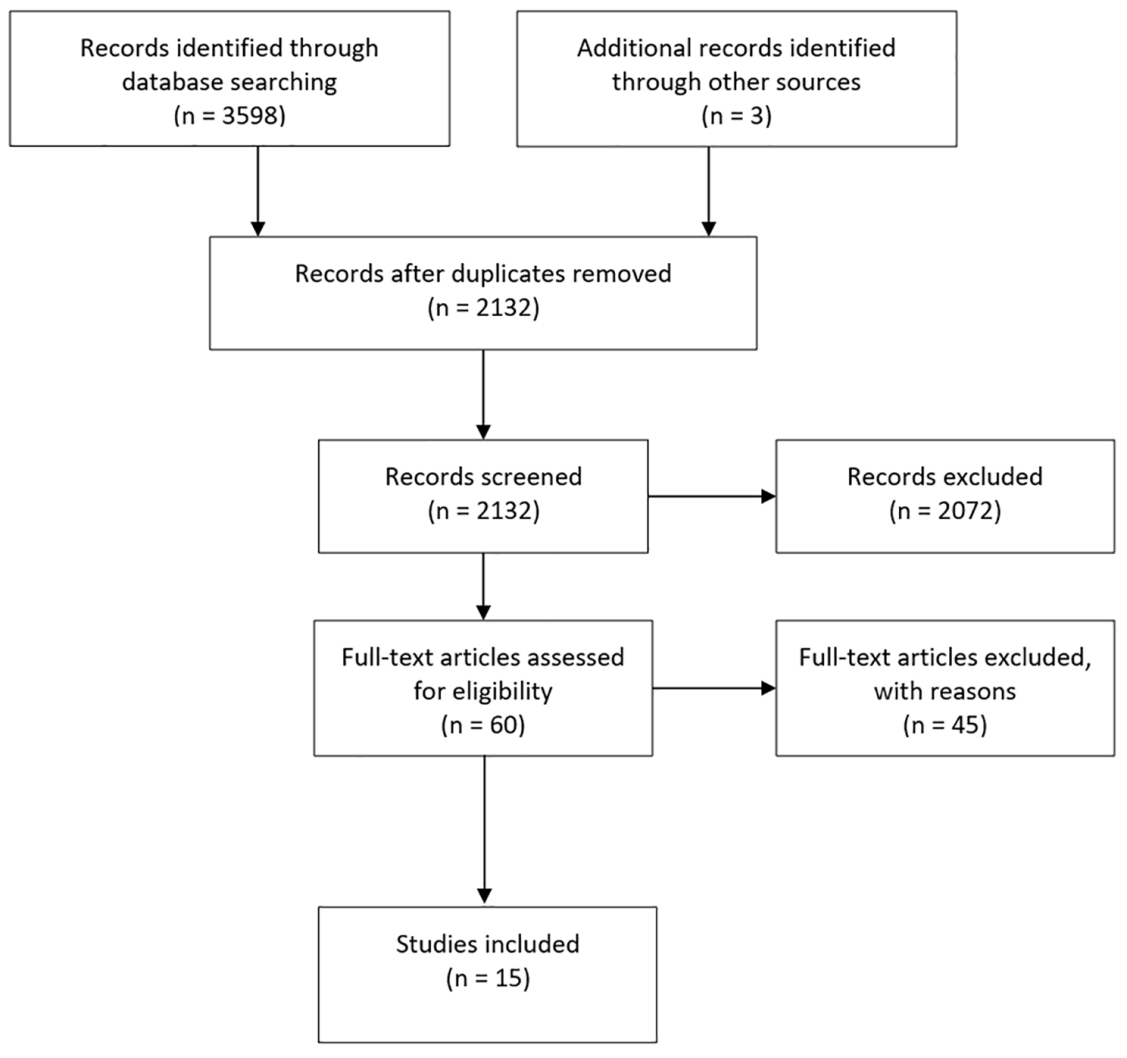
Inclusion flow diagramm

The search strategy identified 2132 individual publications including two additional studies identified through other means (hand search and reference list search) (Fig. 2). 15 studies were included in the final analysis [27–41] (for description of the included studies, please see supplementary file 2). Cohen’s κ was measured to determine the agreement between the two researchers (NR, DD) on 10% of the overall scope, with a substantial agreement at κ = 0.7975.

Table 1 summarizes the identified SDM steps and components described in the existing literature. From the six steps of SDM (Fig. 1), step three – information about treatment options, was most widely described and mentioned within twelve studies. Ten studies reported the means by which information was delivered (decision aid [27, 33, 34] or through HCP [28, 29, 32, 36, 37, 39, 41]) while two studies did not make any clear specifications [31, 40]. A second most widely applied SDM step was the exploration of patient wishes and concerns in the decision-making process (step 4)[27, 29, 32–34, 36, 37]. Description of how the decision was made was described by six studies [29, 34, 36, 38, 39, 41]. Four studies reported about patient’s goals of care regarding immediate care outcomes which can be attributed to the second step of SDM – the key message [30, 35, 38, 40].

**Table 1:** Identified SDM steps

Author, Year	Aim of the study			Identified SDM steps [Number represents the SDM Step as depicted in Fig. 1]		
Quantitative studies						
Korteland, 2017	Assess whether the use of patient decision aids result in an improved quality of decision-making in prosthetic heart valve selection compared with standard preoperative care in patients accepted for aortic and mitral valve replacement.			(Decision for TAVI previously made by heart team) Intervention: 3. Information delivery through patient decision aid online tool: information about and comparison of two options (mechanical / biological heart valve) 4. Patient wishes self-assessed through patient decision aid online tool: exploration of personal feelings and preferences regarding the two options Control: usual care		
Dharmarajan, 2017	Identify patients’ perceptions of their involvement and satisfaction with treatment selection.			(Comparison between SAVR/TAVI (n = 336) group with medical management group (n = 71)) 3. Information: 97% of TAVI/SAVR and 88% of medical management group agreed to strongly agreed that HCP helped better understand their AS. 95% of SAVR/TAVI group and 87% of medical management group agreed to strongly agreed to be given enough information about pros and cons. 5.Decision: 96% of SAVR/TAVI and 85% of medical management group agreed to strongly agreed being involved in the decision		
Anaya, 2019	Pilot a patient decision aid (DA) which compares mechanical to tissue valve replacement before SAVR			Intervention: DA sent by post mail 3. Information: significant higher conceptual and risk probability knowledge scores in patients that reported using the DA 4. Patient concerns: reoperation (slightly reduced concerns compared to control), severe bleeding and anticoagulants 5. Decision: 50% preferred a tissue valve; 40% being unsure Control: no DA 4.Patient concerns: reoperation, severe bleeding, taking anticoagulants 5. Decision: 67% preferred a tissue valve; 25% being unsure		
Coylewright, 2020	Determine whether the repeated use of a decision aid (DA) by heart teams is associated with greater SDM, along with improved patient-centered outcomes and clinician attitudes about DAs.			Intervention: Repeated use of DA 3. Information delivery by means of decision aids (comparison of TAVI and medical management) 4. Patient wishes: in 41% of cases the elicitation of values and preferences was made by asking questions similar to “What matters most?” Control: Visits without DA		
Schmied, 2015	To determine which information sources and decision criteria are important to patients prior to aortic valve surgery.			3. Information delivery through HCP (no differentiation between pros and cons of various treatment options reported) 4. Patient wishes: improvement of quality of life and prolongation of life		
Korteland, 2015	Assess among adult patients accepted for aortic valve replacement: (1) experience with current clinical decision-making regarding prosthetic valve selection, (2) preferences for SDM and risk presentation and (3) prosthetic valve knowledge and numeracy.			3. Information about options: 59% felt they had sufficient knowledge about two options (biological or mechanical valve)		
Sugiura, 2022	Evaluate elderly symptomatic severe AS patients’ perspectives on their treatment goals and identify factors that influence their treatment choice.			2. Goal of care: “What do you hope to accomplish by your treatment?“ 77.6% aimed to reduce symptom burden; 68.4% aimed to maintain independence; 62.2% aimed to regain the ability to engage in a specific activity; 58.2% aimed for an improvement of prognosis. 5. Decision: 54.1% reported making the decision based on their values; 52% made a decision based on the wish not to become a burden for the family and 34.7% did not want to become a burden for the society		
Bryssinck, 2021	Examine post hoc patient satisfaction and the decision-making process of choosing a prosthesis for aortic valve replacement			3. Information about options: 79.6% felt they were well informed to support their valve choice 4. Patient wishes: 48.7% of the patients believed it is important to be involved in the valve choice 5. Decision: 64.5% of patients stated that the decision was made mainly or only by the HCP and 35.4% stated that the decision was shared between HCP and patient		
Qualitative studies						
Skaar, 2017		Explore conditions for an autonomous choice experienced by older adults who recently underwent TAVR, with a special focus on relational and cognitive aspects.	3. Information delivery through HCP: only communication of risks in case of no intervention 4. Patient wishes: fear of declining/improvement of quality of life 5. Decision: patients reported making the decision on their own; some reported obligation towards relatives to accept treatment recommendation (TAVI)			
Coylewright, 2016		Elicit and report patient-defined goals from elderly patients facing treatment decisions for severe AS.	2. Goals of care: “What do you hope to accomplish by having your valve replaced?“ (i) maintaining independence; (ii) staying alive; (iii) reducing/ eliminating pain or symptoms; and (iv) ability to do a specific activity			
Olsson, 2016		Describe the decision-making process about undergoing TAVI treatment among people with severe aortic stenosis.	3. Information: 8 patients reported feeling ambivalent (unsure about the diagnosis, benefits or effects of TAVI as well as method)			
Beishuizen, 2021		Assess patient expectations and goals before TAVI, and determine after treatment whether they had been met.	2. Goals of care: “What do you hope to accomplish by undergoing this treatment?“ 48.5% aimed to regain the ability to engage in a specific activity; 27.9% aimed to reduce symptom burden; 11.8% aimed to maintain independence.			
Ingle, 2021		Determine decision needs among patients with symptomatic AS	3. Information: Patients seeked information about treatment options 4. Patient wishes: Patients expressed experiencing fear as a consequence of not having enough information or because of illness outcomes. 5. Decision: some patients (number n.a.) expressed the importance of including significant others in deliberating about treatment options. Others expressed the preference to leave the decision to the clinician.			
Picou, 2022		Outline the patient experiences related to AS diagnosis, treatment decisions, self-management, and overall personal feelings and psychological impact of the disease.	3. Information: Patients expressed relying on their HCP to gather information for the decision-making process. Patients also searched for additional information elsewhere. 4. Patient wishes: Patients reported making decisions based on their own preferences			
Col, 2022		Identify, prioritize, and organize patient-reported goals and features of treatment for severe AS	2. Treatment goals: “What are the specific goals that you think are most important to consider when deciding about treating AS?“: have trust in HCP; receive good information; live a long life; reduce future risks; improve quality of life; reduce decisional regret; be independent. 3. Information: 92.6% of the patients rated information about options when replacing or repairing a valve as very important; 85.3% rated information about intra and post operative risks as very important 4. Patient wishes: 65.8% rated a treatment recommendation from the HCP as very important			

ACP on the other hand, or its important components have not been identified as an issue of observation. The fundamental goals of care reported by some studies were related to the outcome of the particular treatment outcome (SAVR, TAVI or medication) but with no evidence of deliberation of fundamental goals of care in case of emergencies or complications during or after the intervention.

### Patient wishes, concerns and preferences regarding decision-making and treatment process

Improvement of quality of life through maintaining independence, ability to perform specific activity and symptom mitigation [29–32, 35, 38, 40] as well as life prolongation [31, 32, 35, 37, 38, 40] were the most often reported patient wishes. Lifelong use of anticoagulants, risk of bleeding or blood clot, valve sound, need for reoperations [28, 34, 37], valve lifespan [32] or other risks like stroke, blood transfusion, prolonged need for ventilations and possibility of dialysis [30] were some of the main patient concerns that were reported in the included studies. Becoming a burden to the family or society was also a concern some patients expressed [38]. Further patient concerns were related to the diagnosis, intervention and its benefits, as well as whether the HCPs medical skills are sufficient to ensure a good outcome [31, 40]. Prognostic information describing the gradual increase of symptoms as well as illness severity (especially when the illness symptoms worsen) led to preferences for an intervention (TAVI) [29, 31]. Comorbidities, on the other hand, led to preferences for palliative care [30].

Low patient literacy regarding the intervention, its benefits and risks, were also reported [28], potentially indicating poor communication between the HCP and patient. Patient literacy improved with the increase of decision aids (DA) [33, 34] use as well as with an increased clinician experience in using DA [33]. The effect of DAs on decisional conflict seems to be contradictory, with some studies reporting no effect [27, 33], and another study reporting a significant effect [34]. This may be explained by the low amount of participants as well as the use of different decisional conflict measurement tools.

### Patient wishes, concerns and preferences regarding support

Studies reported that many patients showed a favorable preference for engaging friends or family in the decision-making process [28, 31], which might suggest a degree of willingness to accept their suggestions or expectations. One study reported that patients either wished to involve the significant others in the decision process or exclude them and leave the decision to the HCP [36]. Some studies show that family members often strongly favored the intervention, which in turn influenced patients’ preference towards it [29–31]. HCPs also expressed strong preferences in favor of the intervention, with some convincing [29], recommending [31] or deciding for the patient [27, 28, 32]. Studies also reported that patients engaged themselves in the decision-making when actively encouraged by their HCPs [29, 30].

### Patient engagement in the decision making process

A few studies reported that some patients expressed an explicit wish to make decisions by themselves [29] or wanted to be involved in the decision-making [28, 31, 39]. One study reported patients making treatment decisions based on their preferences as well as information regarding outcomes like anticoagulation, future interventions and recovery time [37]. Younger patients assessed themselves as being more engaged in the decision-making than the older patients [31–33].

Decisional conflict was mostly reported in pre-operative patients [27, 28, 31], which was lower in patients that trusted their HCPs [29, 31] or that involved their friends and family [28, 31]. Post-operative patients reported a lower decisional conflict [28, 32]. This variation can be explained by the success of the intervention, being better measurable in patients without complications, who survived the intervention. Another study reported that involvement of patients in the decision making process as well as providing enough information significantly decreased the risk of decisional conflict in patients [39]. One study comparing SAVR/TAVI patients to medical management patients observed a difference in decisional regret. 97% of the SAVR/TAVI group agreed to strongly agreed that the decision was the right one as compared to only 69% of medical management group [41].

Cognitive impairment was reported to trigger a passive position in patients during the decision-making process [29].

### SDM and ACP Model

**Fig. 3:**
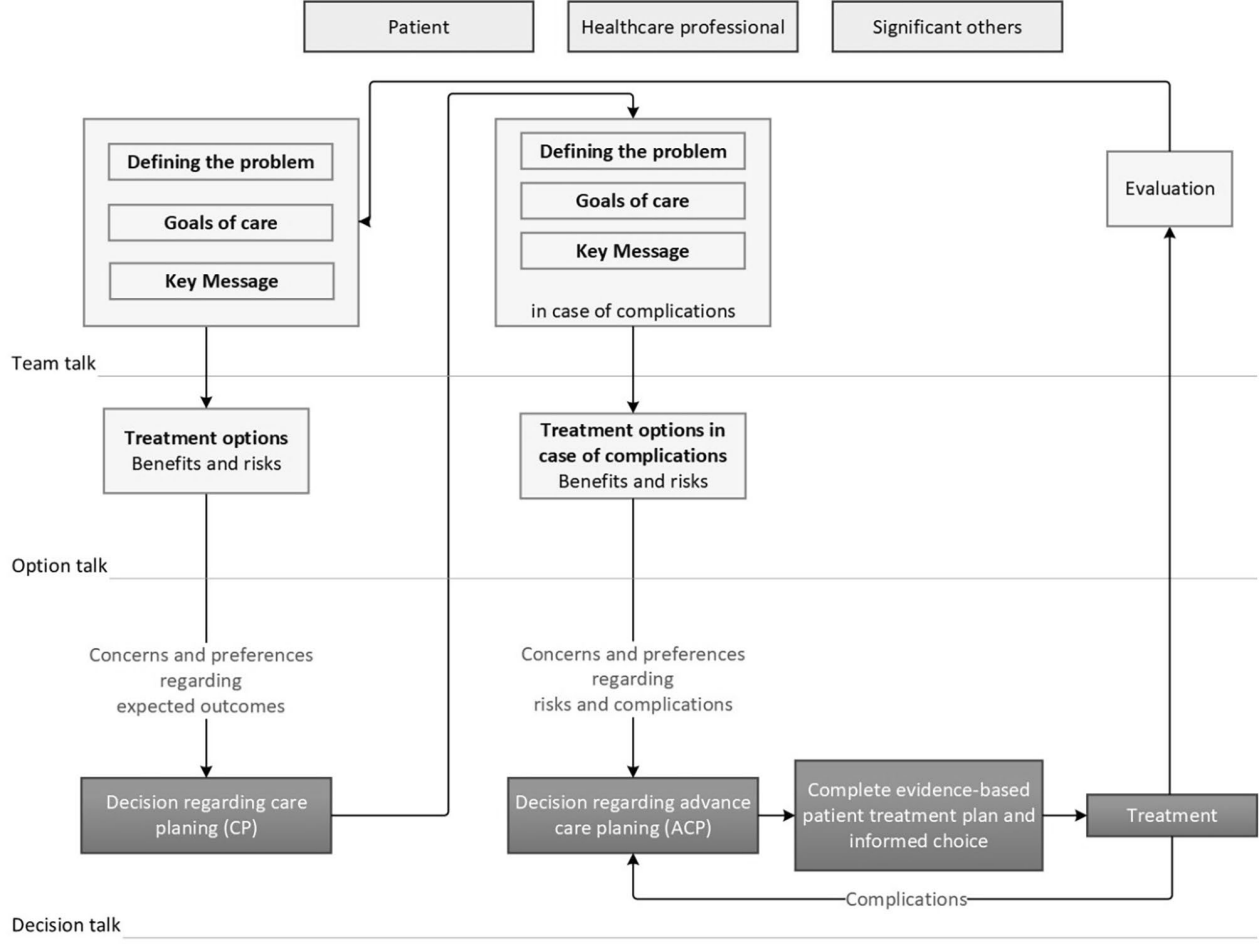
Decision-making process model

To make suggestions for improvement, we built our analysis on combining abductive reasoning with empirics (in this case the results provided by the review). This technique allows us to connect the existing gaps and build new understandings and concepts that may complement the ones more broadly used. We therefore propose a decision-making process, based on the available guidelines and recommendations and the theories on which SDM and ACP are built as well as on the results of this literature review (Fig. 3).

Figure 3 builds upon the SDM model developed by Elwyn et al. [10, 42] and on the six SDM components described by Makoul et al. [13] and Bomhof-Roordink et al. [14].

### Team talk

The SDM scientific community has historically focused on a bidirectional communication process between the patient and the HCP. ACP, on the other hand, has explicitly encouraged the inclusion of significant others (family or close friends) in the decision-making process. As reported in the included studies, the patients with AS were willing to involve their loved ones in the decision-making process. The model proposes to create a team made out of important decision-makers – the patient, their significant others (family and/or friends) and HCPs. Here “HCPs” would include the entire treating team – general practitioner, cardiologist, surgeon, study nurse and whenever needed, a geriatrist and palliative care team.

The decision-making team will proceed by defining the problem that must be addressed (AS management), discussing the patient’s disease/symptom goals of care for the immediate treatment and make sure there is common understanding that the decision should be based both on evidence and patient’s own preferences and wishes (key message or choice awareness).

### Option talk

At this stage, the HCPs present the necessary information and evidence regarding all available treatment options. The benefits and risks of each option (TAVI, SAVR, palliative care), including the option of “wait and see” should be discussed. The HCP should also explain the quality of the evidence and its sources, going on to differentiate between own experiences or observations and current peer-reviewed literature. This will help the patient and their significant others balance out risks against the benefits. Ideally, the HCP will use a validated decision aid to help the patient better compare the available options.

### Decision talk

The decision talk between the patient, their significant other and HCPs are best made in iterative manner. At first, the concerns, preferences and expectations regarding the treatment outcomes are openly expressed by the patient, their significant other and is requested, the HCPs. If the patient is ready, they may make a decision regarding their immediate care planning – TAVI, SAVR or palliative care. They may also decide to leave or delegate decisions to their significant others. If, for example, the patient decides to choose TAVI, the entire team will then initiate an ACP team talk in case of complications. This will mark the beginning of the ACP process.

### ACP Team Talk

At this stage, the decision-makers discuss how the patient wishes to be treated in case of complications that may occur (in this case after a TAVI intervention), or if their health condition deteriorates and the patient is not able to make autonomous decisions anymore. According to the existing ACP models [43], special guiding questions can be helpful to formulate preferences regarding fundamental and functional goals of care for different situations of incapacity of decision making (emergency, ICU treatment with risk for preference-sensitive outcomes, permanent incapability of decision-making etc.).

### ACP option talk

Depending on the treatment risks of complications, different treatment options may be considered (resuscitation during the intervention, intensive care, etc.) All of these carry their own risks and should be discussed during the ACP option talk process. Ideally, the HCP will use a validated decision aid to help the patient better compare the available options.

### ACP decision talk

During the ACP decision talk, the team should express their preferences and concerns regarding the discussed goals of care in case of complications, also making decisions and document choices regarding advance care planning.

### Evidence-based care planning and advance care planning

Once the patient has decided on a care plan and an advance care plan, one could conclude that a complete evidence-based patient treatment plan and informed choice has been made. At this point, it is particularly important to document all patient care and advance care preferences in the form of informed consent and advance directives, or an equal documentation of patient’s preferences in the case of possible emergencies and complications, accessible by surgeons, cardiologists, anesthesiologists and ICU or palliative care physicians alike. After the decision has been implemented (the TAVI was performed), the patient may re-evaluate their decisions and make adjustments where needed.

## Discussion

Patients with aortic stenosis may experience deterioration of their condition at any time. In addition, although therapies such as SAVR or TAVI can offer symptom relief, they can cause complications. By means of SDM and decision aids [44], patients can align their preferences to the existing treatment options (TAVI, SAVR or medication only, targeting life prolongation and/or quality of life and symptoms). SDM allows for broad information exchange based on evidence-based medicine. Patient treatment preferences can also be prediscussed for such situations with complications. This needs a systematic discussion of the fundamental goals of care in these scenarios according to the concept of ACP. ACP supports the patient to make plans regarding their treatment before the occurrence of a future sudden, prolonged or chronic incapacity of decision-making. Historically, SDM was developed to be used in prevention or acute care settings for cases with reduced medical complexity. As SDM proved its value for facilitating patient autonomy, it has been increasingly used in more complex situations which involve chronic illnesses as well as in situations in which a treatment decision might imply risky treatment complications (as in the case of patients for AS). To be able to ensure that autonomous patient treatment choices are being integrated in the treatment process in cases of decision-making incapacity, it is advisable to extend SDM into a broader process, which includes ACP.

The novelty of this paper is the practical merger of the two concepts to improve decision-making processes during care planning and advance care planning. It allows participating decision-makers to have a better understanding about the short- and long-term treatment options, as well as the patient’s short- and long-term goals of care. This approach aims at supporting patients to make better informed decisions for immediate care, as well as to better prepare for future care in case expected treatment outcomes differ from the ones expected in case of a successful intervention. This may help patients and their significant others build more accurate expectations about their treatment and recovery path, therefore helping better manage this stressful situation. Integrating SDM and ACP into one complex process may seem costly and time consuming at first, however, an interdisciplinary approach may help better manage resources to offer best support for patients while keeping costs and time needed for decision deliberation lower than if these issues had not been discussed with the patient himself/herself before the intervention [45]. Strong collaborative relationships between treating HCPs and advance practice nurses or nursing experts may help break the decision-making process in meaningful blocks managed by different experts belonging to one treating team [46]. Therefore, a patient with AS may perform the SDM process regarding the immediate care of AS together with their significant others, a cardiologist, a heart surgeon, a geriatrist and their general practitioner. The decision-making process may afterwards be continued by a cardiology nursing expert with further training in ACP to determine how the patient wants to continue their treatment in case of inability to make decisions for themselves after the intervention and to discuss preferences in case of unforeseen emergencies. At this stage, the treating HCPs may decide to not participate in the ACP decision-making process, but rather take knowledge of the patient’s advance directives, which result from the process.

### Limitations

Because of the rather limited number of studies documenting the implementation of SDM and ACP in current care, this review allows us to draw conclusions with limited depth and representativeness about decision-making in severe AS patients. The ten included studies with post intervention data collection [28, 29, 32, 35–41] might bias the results of this review, as their sample most probably excluded patients that decided for palliative care, or were incapacitated by the intervention or were deeply unsatisfied with the treatment outcome, which might have had an impact on their willingness to continue participating in the study.

The lack of consensus on the exact content and application of SDM and ACP makes it difficult to make clear assumption regarding the use and implementation of the two concepts in the existing literature.

The integrative model described in Fig. 3 may only be used in situations in which patients can make autonomous treatment decisions. The model cannot be implemented in decision-making processes in which patients have already lost the decision-making capacity.

### Solutions

Further research on the implementation of current guidelines to integrate SDM and ACP in usual care is highly recommended. For such research to be able to take place, the SDM and ACP scientific community must agree on o validated SDM and ACP standards for chronically ill or risks of treatment/intervention complications. These should be complemented with studies researching the barriers and facilitators towards better engaging patients in their own care. A longitudinal prospective cohort study detailing the implementation of SDM and ACP in patients with complicative outcomes (like severe AS patients) could better describe the exact implementation processes, its effects as well as possible barriers and facilitators. Development of decision aids which combines fundamental and disease/symptom goals of care for immediate outcomes (SDM) as well as for situations in which patients might lose their decision-making capacity (ACP) could help determine how they perform compared to decision aids that only focus on immediate outcomes (SDM). Another important field of research would involve the use of goals of care formulated for immediate outcomes (SDM) and future outcomes in case of decision making incapacity (ACP), and how they impact the decisional regret and emotional burden of surrogates or other significant others also involved in the decision-making process as compared to surrogates that only participated in goals of care deliberations for immediate outcomes (SDM).

## Conclusion

SDM and ACP are similar concepts with an identical aim – to ensure that patients receive the treatment which best aligns with their preferences, values and short- and long-term goals of care. SDM mostly focuses on the short to mid-term goals of care, while ACP mostly focuses on long-term goals of care in case of hypothetical situations of reduced or limited decision-making capacity. Although both concepts can be applied separately, they must be integrated in the case of patients, whose treatment decisions might lead to loss of decision-making capacity during or shortly after an intervention.

When individuals become severely ill, patient autonomy may become very fragile and subject to various influences stemming from the patient themselves or from the outside. Illness burden, HCP or family members’ preferences are strong predictors of the patient’s final decision. An iterative process allows for each important decision-maker to share their preferences and concerns regarding the care planning and advance care planning. This would help to better structure and prioritize information while creating a trustful and respectful relationship between the participants. This may support not only patients to make autonomous decisions, but also allow for family members to best support them, and HCPs to deliver care which best complies with their patient’s values and fundamental goals of care. This ongoing support can provide the basis for enabling patient autonomy throughout the treatment process.

### Electronic supplementary material

Below is the link to the electronic supplementary material.

Additional File 1: Cochrane library

Additional File 2: Summary of included studies

Additional File 3: PRISMA 2020 checklist

## Data Availability

The data used for this review can be found in the supplementary file 1 and 2. Further information is available from the corresponding author. Legends:
