# Supplementary material for: Shared decision making and advance care planning: a systematic literature review and novel decision-making model
Source: BMC Med Ethics. 2023 Aug 14;24:64. doi: 10.1186/s12910-023-00944-7 (PMC10426137; doi:10.1186/s12910-023-00944-7)
Supplement: Supplementary file 1 — Additional File 1: Cochrane library [file 12910_2023_944_MOESM1_ESM.pdf]

Cookies
Our site uses cookies to improve your experience. You can find out more about our use of cookies in About Cookies, including instructions on how to turn off cookies if you wish to do so. By continuing to browse this site you agree to us using cookies as described in About Cookies.

I accept

Cochrane Library

Access provided by: **UZH Hauptbibliothek / Zentralbibliothek Zürich**

Advanced Search

Search manager

Save this search | View saved searches | ? Search help

View fewer lines | Print

#1	((((heart or cardiac OR aort*) NEAR/3 (stenos*)) OR (valv* NEAR/3 (disease* OR dysfunction* OR failure OR replac* OR prosthes*)) OR (TAVR OR transcatheter OR transfemoral OR transapical OR transaxillary OR SAVR OR "surgical AVR" OR TAVI)) AND ((aged OR older OR elder* OR geriatric*))):ti,ab,kw<br>(Word variations have been searched)	3759
#2	((shar* OR joint OR together OR consensual OR collaborativ* OR cooperativ* OR mutual* OR united OR combined OR conjoint* OR accept* OR informed) NEXT/3 decision*):ti,ab,kw OR ((patient* OR consumer* OR famil* OR personal) NEXT/3 (decision* OR choice OR judg* OR consent* OR consens* OR assent* OR dissent* OR preference* OR agreement OR disagreement OR preference* OR involv*)):ti,ab,kw	19424
#3	((decision OR choice):ti,ab,kw) AND (((doctor OR physician OR professional* OR surgeon) NEAR/3 (patient OR consumer OR family) NEAR/3 (relation* OR communication OR interview OR discussion OR consultation)):ti,ab,kw OR ((interpersonal OR personal OR human) NEAR/3 (relation* OR communicat*)):ti,ab,kw OR ((health OR illness) NEAR/3 (attitude* OR knowledge OR education OR information OR literacy OR behavior OR behaviour)):ti,ab,kw OR ((choice OR cooperative OR co-operative) NEAR/3 (behavior OR behaviour)):ti,ab,kw OR ((patient OR consumer) NEAR/3 (attitude* OR participation* OR refusal OR	7828

adherence OR compliance OR nonadherence OR non-adherence OR education OR acceptance OR cooperation OR co-operation OR collaboration* OR information OR misinformation OR education OR disput* OR involv*))):ti,ab,kw

#4	#2 OR #3	Limits	24923
#5	#1 AND #4	Limits	74
#6	(((advance* OR future OR prospect* OR forthcoming) NEXT/3 (care OR treatment OR therapy) NEXT/3 (plan* OR intention* OR scheme* OR schedule* OR arrangement* OR measure* OR step* OR intervention* OR procedure* OR action*)):ti,ab,kw) OR (((advance* OR future OR prospect* OR forthcoming) NEXT/3 (directive* OR instruction* OR preferences) OR (living NEAR/3 will*)):ti,ab,kw)	Limits	1342
#7	#1 AND #6	Limits	0
#8	#5 OR #7	Limits	74
#9	Type a search term or use the S or MeSH	S MeSH Limits	N/A

Clear all

Highlight orphan lines

Save this search | View saved searches | Search help

View fewer lines | Print

Wednesday, January 13, 2021 5:46:54 AM

#	Query	Limiters/Expanders	Last Run Via	Results
S8	S5 OR S7	Expanders - Apply equivalent subjects Search modes - Find all my search terms	Interface - EBSCOhost Research Databases Search Screen - Advanced Search Database - CINAHL with Full Text	99
S7	S1 AND S6	Expanders - Apply equivalent subjects Search modes - Find all my search terms	Interface - EBSCOhost Research Databases Search Screen - Advanced Search Database - CINAHL with Full Text	5
S6	(MH "Advance Care Planning") OR (MH "Advance Directives+") OR (MH "Living Wills") OR TI ((advance* OR future OR prospect* OR forthcoming) W3 (care OR treatment OR therapy) W3 (plan* OR intention* OR scheme* OR schedule* OR arrangement* OR measure* OR step* OR intervention* OR procedure* OR action*)) OR TI ((advance* OR future OR prospect* OR forthcoming) W3 (directive* OR instruction* OR preferences)) OR TI (living N1 will*) OR AB ((advance* OR future OR prospect* OR forthcoming) W3 (care OR treatment OR therapy) W3 (plan* OR intention* OR scheme* OR schedule*	Expanders - Apply equivalent subjects Search modes - Find all my search terms	Interface - EBSCOhost Research Databases Search Screen - Advanced Search Database - CINAHL with Full Text	14,172

	OR arrangement* OR measure* OR step* OR intervention* OR procedure* OR action*)) OR AB ((advance* OR future OR prospect* OR forthcoming) W3 (directive* OR instruction* OR preferences)) OR AB (living N1 will*)			
S5	S1 AND S4	Expanders - Apply equivalent subjects<br>Search modes - Find all my search terms	Interface - EBSCOhost Research Databases<br>Search Screen - Advanced Search<br>Database - CINAHL with Full Text	94
S4	S2 OR S3	Expanders - Apply equivalent subjects<br>Search modes - Find all my search terms	Interface - EBSCOhost Research Databases<br>Search Screen - Advanced Search<br>Database - CINAHL with Full Text	109,976
S3	((MH "Decision Making+") OR (MH "Decision Making, Computer Assisted+") OR (MH "Decision Support Systems, Clinical") OR (MH "Decision Support Techniques") OR TI (decision OR choice) OR AB (decision OR choice)) AND ((MH "Patient Compliance+") OR (MH "Patient Participation") OR (MH "Attitude to Health+") OR (MH "Professional-Patient Relations+") OR (MH "Illness Behavior") OR (MH "Interpersonal Relations+") OR (MH "Interdisciplinary Communication") OR TI ((doctor OR physician OR	Expanders - Apply equivalent subjects<br>Search modes - Find all my search terms	Interface - EBSCOhost Research Databases<br>Search Screen - Advanced Search<br>Database - CINAHL with Full Text	57,793

professional* OR surgeon) N3 (patient OR consumer OR family) N3 (relation* OR communication OR interview OR discussion OR consultation)) OR TI ((interpersonal OR personal OR human) N3 (relation* OR communicat*)) OR TI ((health OR illness) N3 (attitude* OR knowledge OR education OR information OR literacy OR behavior OR behaviour)) OR TI ((choice OR cooperative OR cooperative) N3 (behavior OR behaviour)) OR TI ((patient OR consumer) N3 (attitude* OR participation* OR refusal OR adherence OR compliance OR nonadherence OR nonadherence OR education OR acceptance OR cooperation OR co-operation OR collaboration* OR information OR misinformation OR education OR disput* OR involv*)) AB ((doctor OR physician OR professional* OR surgeon) N3 (patient OR consumer OR family) N3 (relation* OR communication OR interview OR discussion OR consultation)) OR AB ((interpersonal OR personal OR human) N3 (relation* OR communicat*)) OR AB ((health OR illness) N3

	(attitude* OR knowledge OR education OR information OR literacy OR behavior OR behaviour)) OR AB ((choice OR cooperative OR cooperative) N3 (behavior OR behaviour)) OR AB ((patient OR consumer) N3 (attitude* OR participation* OR refusal OR adherence OR compliance OR nonadherence OR nonadherence OR education OR acceptance OR cooperation OR co-operation OR collaboration* OR information OR misinformation OR education OR disput* OR involv*)))			
S2	(MH "Decision Making, Shared") OR (MH "Decision Making, Patient+") OR (MH "Patient Preference") OR (MH "Decision Making, Family") OR TI ((shar* OR joint OR together OR consensual OR collaborativ* OR cooperativ* OR mutual* OR united OR combined OR conjoint* OR accept* OR informed) W3 decision*) OR TI ((patient* OR consumer* OR famil* OR personal) W3 (decision* OR choice OR judg* OR consent* OR consens* OR assent* OR dissent* OR preference* OR agreement OR disagreement OR	Expanders - Apply equivalent subjects<br>Search modes - Find all my search terms	Interface - EBSCOhost Research Databases<br>Search Screen - Advanced Search<br>Database - CINAHL with Full Text	70,704

	preference* OR involv*)) OR AB ((shar* OR joint OR together OR consensual OR collaborativ* OR cooperativ* OR mutual* OR united OR combined OR conjoint* OR accept* OR informed) W3 decision*) OR AB ((patient* OR consumer* OR famil* OR personal) W3 (decision* OR choice OR judg* OR consent* OR consens* OR assent* OR dissent* OR preference* OR agreement OR disagreement OR preference* OR involv*))			
S1	((MH "Aortic Valve Stenosis+") OR (MH "Aortic Valve Insufficiency") OR (MH "Heart Valve Prosthesis") OR TI ((heart or cardiac OR aort*) N3 (stenos*)) OR TI (valv* N3 (disease* OR dysfunction* OR failure OR replac* OR prosthes*)) OR TI (TAVR OR transcatheter OR transfemoral OR transapical OR transaxillary OR SAVR OR "surgical AVR" OR TAVI) OR AB ((heart or cardiac OR aort*) N3 (stenos*)) OR AB (valv* N3 (disease* OR dysfunction* OR failure OR replac* OR prosthes*)) OR AB (TAVR OR transcatheter OR transfemoral OR transapical OR transaxillary OR SAVR	Expanders - Apply equivalent subjects<br>Search modes - Find all my search terms	Interface - EBSCOhost Research Databases<br>Search Screen - Advanced Search<br>Database - CINAHL with Full Text	7,999

OR "surgical AVR" OR TAVI)) AND ((MH "Aged+") OR (MH "Health Services for the Aged") OR (MH "Geriatric Assessment+") OR (MH "Geriatrics") OR TI (aged OR older OR elder* OR geriatric*) OR AB (aged OR older OR elder* OR geriatric*))

No.,Query,Results,Date
#8,"#5 OR #7",663,13 Jan 2021
#7,"#1 AND #6",10,13 Jan 2021
#6,"'advance care planning'/exp OR 'living will'/exp OR (((advance* OR future OR prospect* OR forthcoming) NEXT/3 (care OR treatment OR therapy) NEXT/3 (plan* OR intention* OR scheme* OR schedule* OR arrangement* OR measure* OR step* OR intervention* OR procedure* OR action*)):ti,ab) OR (((advance* OR future OR prospect* OR forthcoming) NEXT/3 (directive* OR instruction* OR preferences)):ti,ab) OR ((living
#5,"#1 AND #4",653,13 Jan 2021
#4,"#2 OR #3",276853,13 Jan 2021
#3,"('decision making'/exp OR 'decision support system'/exp OR decision:ti,ab OR choice:ti,ab) AND ('patient attitude'/exp OR 'doctor patient relationship'/exp OR 'patient compliance'/exp OR 'attitude to health'/exp OR 'illness behavior'/exp OR 'interpersonal communication'/exp OR (((doctor OR physician OR professional* OR surgeon) NEAR/3 (patient OR consumer OR family) NEAR/3 (relation* OR communication OR interview OR discussion OR consultation)):ti,ab) OR (((interpersonal OR personal OR human) NEAR/3 (relation* OR communicat*)):ti,ab) OR (((health OR illness) NEAR/3 (attitude* OR knowledge OR education OR information OR literacy OR behavior OR behaviour)):ti,ab) OR (((choice OR cooperative OR 'co operative') NEAR/3 (behavior OR behaviour)):ti,ab) OR (((patient OR consumer) NEAR/3 (attitude* OR participation* OR refusal OR adherence OR
#2,"'shared decision making'/exp OR 'patient preference'/exp OR 'family decision making'/exp OR 'patient decision making'/exp OR (((shar* OR joint OR together OR consensual OR collaborativ* OR cooperativ* OR mutual* OR united OR combined OR conjoint* OR accept* OR informed) NEXT/3 decision*):ti,ab) OR (((patient* OR consumer* OR famil* OR personal) NEXT/3 (decision* OR choice OR judg* OR consent* OR consens* OR
#1,"('aortic valve disease'/exp OR 'aortic valve disease' OR 'aortic stenosis'/exp OR 'aortic stenosis' OR 'aortic valve stenosis'/exp OR 'aortic valve stenosis' OR 'aortic valve replacement'/exp OR 'aortic valve replacement' OR 'aortic valve prosthesis'/exp OR 'aortic valve prosthesis' OR (((heart OR cardiac OR aort*) NEAR/3 stenos*):ti,ab) OR ((valv* NEAR/3 (disease* OR dysfunction* OR failure OR replac* OR prosthes*)):ti,ab) OR tavr:ti,ab OR transcatheter:ti,ab OR transfemoral:ti,ab OR transapical:ti,ab OR transaxillary:ti,ab OR savr:ti,ab OR 'surgical avr':ti,ab OR tavi:ti,ab) AND ('aged'/exp OR 'aged' OR 'geriatrics'/exp OR 'geriatrics' OR 'geriatric assessment'/exp

Brought to you by UZH Hauptbibliothek / Zentralbibliothek Zürich

Saved searches

Combine queries... e.g. #1 AND NOT #3

ID	Name	Query	Documents	Date last run	Actions
#1	heart cardiac aort stenos valv	(((TITLE-ABS-KEY ((heart OR cardiac OR aort*) W/3 (stenos *)) OR TITLE-ABS-KEY (valv* W/3 (disease* OR dysfunction* OR failure OR replac* OR pr osthes*)) OR TITLE-ABS-KEY (tavr OR transcatheter OR trans femoral OR transapical OR tra nsaxillary OR savr OR "surgical AVR" OR tavi)) AND (TITLE-A BS-KEY (aged OR older OR el der* OR geriatric*))) AND ((TITLE-ABS-KEY ((shar* OR joi nt OR together OR consensual OR collaborativ* OR cooperati v* OR mutual* OR united OR combined OR conjoint* OR a ccept* OR informed) PRE/3 d ecision*) OR TITLE-ABS-KEY ((patient* OR consumer* OR fa mil* OR personal) PRE/3 (dec ision* OR choice OR judg* O R consent* OR consens* OR assent* OR dissent* OR prefer ence* OR agreement OR disag reement OR preference* OR i nvolv*))) OR ((TITLE-ABS-KE Y (decision OR choice)) AND (TITLE-ABS-KEY ((doctor OR physician OR professional* OR surgeon) W/3 (patient OR co nsumer OR family) W/3 (relat ion* OR communication OR i nterview OR discussion OR co nsultation)) OR TITLE-ABS-KE Y ((interpersonal OR personal OR human) W/3 (relation* O R communicat*)) OR TITLE-A BS-KEY ((health OR illness) W/3 (attitude* OR knowledge OR education OR information OR literacy OR behavior OR b ehaviour)) OR TITLE-ABS-KEY ((choice OR cooperative OR c o-operative) W/3 (behavior OR behaviour)) OR TITLE-ABS-K EY ((patient OR consumer) W/3 (attitude* OR participatio n* OR refusal OR adherence OR compliance OR nonadhere nce OR non-adherence OR ed ucation OR acceptance OR coo peration OR co-operation OR collaboration* OR information OR misinformation OR educati	908	14 Jan 2021	

ID	Name	Query	Documents	Date last run	Actions
		on OR disput* OR involv*))))) OR (((TITLE-ABS-KEY ((heart OR cardiac OR aort*) W/3 (stenos*)) OR TITLE-ABS-KEY (valv* W/3 (disease* OR dysfunction* OR failure OR replac* OR prosthes*)) OR TITLE-ABS-KEY (tavr OR transcatheter OR transfemoral OR transapical OR transaxillary OR savr OR "surgical AVR" OR tavi)) AND (TITLE-ABS-KEY (aged OR older OR elder* OR geriatric*))) AND ((TITLE-ABS-KEY ((advance* OR future OR prospect* OR forthcoming) PRE/3 (care OR treatment OR therapy) PRE/3 (plan* OR intention* OR scheme* OR schedule* OR arrangement* OR measure* OR step* OR intervention* OR procedure* OR action*))) OR (TITLE-ABS-KEY ((advance* OR future OR prospect* OR forthcoming) PRE/3 (directive* OR instruction* OR preferences))) OR (TITLE-ABS-KEY (living PRE/1 will*))))) View Less Edit query			

About Scopus

What is Scopus
Content coverage
Scopus blog
Scopus API
Privacy matters

Language

日本語に切り替える
切换到简体中文
切換到繁體中文
Русский язык

Customer Service

Help
Contact us

ELSEVIER

Terms and conditions ↗ Privacy policy ↗

Copyright © Elsevier B.V ↗. All rights reserved. Scopus® is a registered trademark of Elsevier B.V.
We use cookies to help provide and enhance our service and tailor content. By continuing, you agree to the use of cookies.

RELX

Wednesday, January 13, 2021 5:07:14 AM

#	Query	Limiters/Expanders	Last Run Via	Results
S8	S7 OR S5	Expanders - Apply equivalent subjects Search modes - Find all my search terms	Interface - EBSCOhost Research Databases Search Screen - Advanced Search Database - MEDLINE	566
S7	S1 AND S6	Expanders - Apply equivalent subjects Search modes - Find all my search terms	Interface - EBSCOhost Research Databases Search Screen - Advanced Search Database - MEDLINE	10
S6	(MH "Advance Care Planning+") OR (MH "Advance Directives+") OR (MH "Living Wills") OR TI ((advance* OR future OR prospect* OR forthcoming) W3 (care OR treatment OR therapy) W3 (plan* OR intention* OR scheme* OR schedule* OR arrangement* OR measure* OR step* OR intervention* OR procedure* OR action*)) OR TI ((advance* OR future OR prospect* OR forthcoming) W3 (directive* OR instruction* OR preferences)) OR TI (living N1 will*) OR AB ((advance* OR future OR prospect* OR forthcoming) W3 (care OR treatment OR therapy) W3 (plan* OR intention* OR scheme* OR schedule* OR arrangement* OR measure* OR step* OR	Expanders - Apply equivalent subjects Search modes - Find all my search terms	Interface - EBSCOhost Research Databases Search Screen - Advanced Search Database - MEDLINE	14,887

	intervention* OR procedure* OR action*)) OR AB ((advance* OR future OR prospect* OR forthcoming) W3 (directive* OR instruction* OR preferences)) OR AB (living N1 will*)			
S5	S1 AND S4	Expanders - Apply equivalent subjects<br>Search modes - Find all my search terms	Interface - EBSCOhost Research Databases<br>Search Screen - Advanced Search<br>Database - MEDLINE	558
S4	S2 OR S3	Expanders - Apply equivalent subjects<br>Search modes - Find all my search terms	Interface - EBSCOhost Research Databases<br>Search Screen - Advanced Search<br>Database - MEDLINE	276,579
S3	((MH "Clinical Decision-Making") OR (MH "Decision Support Techniques+") OR (MH "Decision Support Systems, Clinical") OR TI (decision OR choice) OR AB (decision OR choice)) AND ((MH "Patient Compliance+")OR (MH "Patient Participation") OR (MH "Attitude to Health+") OR (MH "Professional-Patient Relations+") OR (MH "Illness Behavior") OR (MH "Interpersonal Relations+") OR (MH "Interdisciplinary Communication") OR TI ((doctor OR physician OR professional* OR surgeon) N3 (patient OR consumer OR family) N3 (relation* OR communication OR interview OR discussion OR consultation)) OR TI	Expanders - Apply equivalent subjects<br>Search modes - Find all my search terms	Interface - EBSCOhost Research Databases<br>Search Screen - Advanced Search<br>Database - MEDLINE	91,631

((interpersonal OR
personal OR human) N3
(relation* OR
communicat*)) OR TI
((health OR illness) N3
(attitude* OR knowledge
OR education OR
information OR literacy
OR behavior OR
behaviour)) OR TI
((choice OR cooperative
OR cooperative) N3
(behavior OR behaviour))
OR TI ((patient OR
consumer) N3 (attitude*
OR participation* OR
refusal OR adherence OR
compliance OR
nonadherence OR
nonadherence OR
education OR acceptance
OR cooperation OR co-
operation OR
collaboration* OR
information OR
misinformation OR
education OR disput* OR
involv*)) AB ((doctor OR
physician OR
professional* OR surgeon)
N3 (patient OR consumer
OR family) N3 (relation*
OR communication OR
interview OR discussion
OR consultation)) OR AB
((interpersonal OR
personal OR human) N3
(relation* OR
communicat*)) OR AB
((health OR illness) N3
(attitude* OR knowledge
OR education OR
information OR literacy
OR behavior OR
behaviour)) OR AB
((choice OR cooperative

	OR cooperative) N3 (behavior OR behaviour)) OR AB ((patient OR consumer) N3 (attitude* OR participation* OR refusal OR adherence OR compliance OR nonadherence OR nonadherence OR education OR acceptance OR cooperation OR co-operation OR collaboration* OR information OR misinformation OR education OR disput* OR involv*)))			
S2	(MH "Decision Making") OR (MH "Patient Preference") OR TI ((shar* OR joint OR together OR consensual OR collaborativ* OR cooperativ* OR mutual* OR united OR combined OR conjoint* OR accept* OR informed) W3 decision*) OR TI ((patient* OR consumer* OR famil* OR personal) W3 (decision* OR choice OR judg* OR consent* OR consens* OR assent* OR dissent* OR preference* OR agreement OR disagreement OR preference* OR involv*)) OR AB ((shar* OR joint OR together OR consensual OR collaborativ* OR cooperativ* OR mutual* OR united OR combined OR conjoint* OR accept* OR informed) W3 decision*) OR AB	Expanders - Apply equivalent subjects<br>Search modes - Find all my search terms	Interface - EBSCOhost Research Databases<br>Search Screen - Advanced Search<br>Database - MEDLINE	220,901

	((patient* OR consumer* OR famil* OR personal) W3 (decision* OR choice OR judg* OR consent* OR consens* OR assent* OR dissent* OR preference* OR agreement OR disagreement OR preference* OR involv*))			
S1	((MH "Aortic Valve Stenosis+") OR (MH "Aortic Valve Insufficiency") OR (MH "Transcatheter Aortic Valve Replacement") OR (MH "Heart Valve Prosthesis") OR TI ((heart or cardiac OR aort*) N3 (stenos*)) OR TI (valv* N3 (disease* OR dysfunction* OR failure OR replac* OR prosthes*)) OR TI (TAVR OR transcatheter OR transfemoral OR transapical OR transaxillary OR SAVR OR surgical AVR OR TAVI) OR AB ((heart or cardiac OR aort*) N3 (stenos*)) OR AB (valv* N3 (disease* OR dysfunction* OR failure OR replac* OR prosthes*)) OR AB (TAVR OR transcatheter OR transfemoral OR transapical OR transaxillary OR SAVR OR surgical AVR OR TAVI)) AND ((MH "Aged+") OR (MH "Health Services for the Aged") OR (MH "Geriatric Assessment") OR (MH "Geriatrics") OR TI (aged OR older OR elder* OR	Expanders - Apply equivalent subjects<br>Search modes - Find all my search terms	Interface - EBSCOhost Research Databases<br>Search Screen - Advanced Search<br>Database - MEDLINE	46,351

geriatric*) OR AB (aged OR older OR elder* OR geriatric*))

MY EBSCOhost

Wednesday, January 13, 2021 6:05:40 AM

#	Query	Limiters/Expanders	Last Run Via	Results
S8	S5 OR S7	Expanders - Apply equivalent subjects<br>Search modes - Find all my search terms	Interface - EBSCOhost Research Databases<br>Search Screen - Advanced Search<br>Database - APA PsycInfo	10
S7	S1 AND S6	Expanders - Apply equivalent subjects<br>Search modes - Find all my search terms	Interface - EBSCOhost Research Databases<br>Search Screen - Advanced Search<br>Database - APA PsycInfo	1
S6	DE "Advance Directives" OR DE "Treatment Refusal" OR DE "Treatment Withholding" OR TI ((advance* OR future OR prospect* OR forthcoming) W3 (care OR treatment OR therapy) W3 (plan* OR intention* OR scheme* OR schedule* OR arrangement* OR measure* OR step* OR intervention* OR procedure* OR action*)) OR TI ((advance* OR future OR prospect* OR forthcoming) W3 (directive* OR instruction* OR preferences)) OR TI (living N1 will*) OR AB ((advance* OR future OR prospect* OR forthcoming) W3 (care OR treatment OR therapy) W3 (plan* OR intention* OR scheme* OR schedule* OR arrangement* OR measure* OR step* OR	Expanders - Apply equivalent subjects<br>Search modes - Find all my search terms	Interface - EBSCOhost Research Databases<br>Search Screen - Advanced Search<br>Database - APA PsycInfo	7,220

	intervention* OR procedure* OR action*)) OR AB ((advance* OR future OR prospect* OR forthcoming) W3 (directive* OR instruction* OR preferences)) OR AB (living N1 will*)			
S5	S1 AND S4	Expanders - Apply equivalent subjects<br>Search modes - Find all my search terms	Interface - EBSCOhost Research Databases<br>Search Screen - Advanced Search<br>Database - APA PsycInfo	10
S4	S2 OR S3	Expanders - Apply equivalent subjects<br>Search modes - Find all my search terms	Interface - EBSCOhost Research Databases<br>Search Screen - Advanced Search<br>Database - APA PsycInfo	90,747
S3	((DE "Decision Making" OR DE "Decision Support Systems") OR TI (decision OR choice) OR AB (decision OR choice)) AND ((MH "Patient Compliance+") OR (MH "Patient Participation") OR (MH "Attitude to Health+") OR (MH "Professional-Patient Relations+") OR (MH "Illness Behavior") OR (MH "Interpersonal Relations+") OR (MH "Interdisciplinary Communication") OR TI ((doctor OR physician OR professional* OR surgeon) N3 (patient OR consumer OR family) N3 (relation* OR communication OR interview OR discussion OR consultation)) OR TI ((interpersonal OR personal OR human) N3 (relation* OR	Expanders - Apply equivalent subjects<br>Search modes - Find all my search terms	Interface - EBSCOhost Research Databases<br>Search Screen - Advanced Search<br>Database - APA PsycInfo	22,210

communicat*)) OR TI ((health OR illness) N3 (attitude* OR knowledge OR education OR information OR literacy OR behavior OR behaviour)) OR TI ((choice OR cooperative OR cooperative) N3 (behavior OR behaviour)) OR TI ((patient OR consumer) N3 (attitude* OR participation* OR refusal OR adherence OR compliance OR nonadherence OR nonadherence OR education OR acceptance OR cooperation OR co-operation OR collaboration* OR information OR misinformation OR education OR disput* OR involv*)) AB ((doctor OR physician OR professional* OR surgeon) N3 (patient OR consumer OR family) N3 (relation* OR communication OR interview OR discussion OR consultation)) OR AB ((interpersonal OR personal OR human) N3 (relation* OR communicat*)) OR AB ((health OR illness) N3 (attitude* OR knowledge OR education OR information OR literacy OR behavior OR behaviour)) OR AB ((choice OR cooperative OR cooperative) N3 (behavior OR behaviour)) OR AB ((patient OR

	consumer) N3 (attitude* OR participation* OR refusal OR adherence OR compliance OR nonadherence OR nonadherence OR education OR acceptance OR cooperation OR co-operation OR collaboration* OR information OR misinformation OR education OR disput* OR involv*)))			
S2	(DE "Choice Behavior" OR DE "Group Decision Making") OR TI ((shar* OR joint OR together OR consensual OR collaborativ* OR cooperativ* OR mutual* OR united OR combined OR conjoint* OR accept* OR informed) W3 decision*) OR TI ((patient* OR consumer* OR famil* OR personal) W3 (decision* OR choice OR judg* OR consent* OR consens* OR assent* OR dissent* OR preference* OR agreement OR disagreement OR preference* OR involv*)) OR AB ((shar* OR joint OR together OR consensual OR collaborativ* OR cooperativ* OR mutual* OR united OR combined OR conjoint* OR accept* OR informed) W3 decision*) OR AB ((patient* OR consumer* OR famil* OR personal) W3 (decision* OR choice	Expanders - Apply equivalent subjects<br>Search modes - Find all my search terms	Interface - EBSCOhost Research Databases<br>Search Screen - Advanced Search<br>Database - APA PsycInfo	76,332

	OR judg* OR consent* OR consens* OR assent* OR dissent* OR preference* OR agreement OR disagreement OR preference* OR involv*))			
S1	((DE "Heart Valves") OR (DE "Aorta") OR (DE "Heart Surgery") OR TI ((heart or cardiac OR aort*) N3 (stenos*)) OR TI (valv* N3 (disease* OR dysfunction* OR failure OR replac* OR prosthes*)) OR TI (TAVR OR transcatheter OR transfemoral OR transapical OR transaxillary OR SAVR OR "surgical AVR" OR TAVI) OR AB ((heart or cardiac OR aort*) N3 (stenos*)) OR AB (valv* N3 (disease* OR dysfunction* OR failure OR replac* OR prosthes*)) OR AB (TAVR OR transcatheter OR transfemoral OR transapical OR transaxillary OR SAVR OR "surgical AVR" OR TAVI)) AND ((MH "Aged+") OR (MH "Health Services for the Aged") OR (MH "Geriatric Assessment+") OR (MH "Geriatrics") OR TI (aged OR older OR elder* OR geriatric*) OR AB (aged OR older OR elder* OR geriatric*))	Expanders - Apply equivalent subjects<br>Search modes - Find all my search terms	Interface - EBSCOhost Research Databases<br>Search Screen - Advanced Search<br>Database - APA PsycInfo	392

Select a database Web of Science Core Collection

Basic Search Author Search BETA Cited Reference Search Advanced Search Structure Search

Use field tags, Boolean operators, parentheses, and query sets to create your query. Results will appear in the Search History table at the bottom of the page.(Learn more about Advanced Search)

Example: TS=(nanotub* AND carbon) NOT AU=Smalley RE
#1 NOT #2 more examples | view the tutorial

Search

Restrict results by languages and document types:

All languages	All document types
English	Article
Afrikaans	Abstract of Published Item
Arabic	Art Exhibit Review

Booleans: AND, OR, NOT, SAME, NEAR

Field Tags:

**TS**= Topic	**SA**= Street Address
**TI**= Title	**CI**= City
**AU**= Author [Index]	**PS**= Province/State
**AI**= Author Identifiers	**CU**= Country/Region
**GP**= Group Author [Index]	**ZP**= Zip/Postal Code
**ED**= Editor	**FO**= Funding Agency
**SO**= Publication Name [Index]	**FG**= Grant Number
**DO**= DOI	**FT**= Funding Text
**PY**= Year Published	**SU**= Research Area
**CF**= Conference	**WC**= Web of Science Category
**AD**= Address	**IS**= ISSN/ISBN
**OG**= Organization-Enhanced [Index]	**UT**= Accession Number
**OO**= Organization	**PMID**= PubMed ID
**SG**= Suborganization	**ALL**= All Fields
**AB**= Abstract	
**AK**= Author Keywords	
**KP**= Keyword Plus ®	

Timespan

All years (1900 - 2021)

More settings

Search History:

Set Results Save History / Create Alert Open Saved History Edit Sets Coml Al Cc

Set	Results	Query	Edit
# 8	827	#5 OR #7<br>*Indexes=SCI-EXPANDED, SSCI, A&HCI, CPCI-S, CPCI-SSH, BKCI-S, BKCI-SSH, ESCI, CCR-EXPANDED, IC Timespan=All years*	Edit
# 7	25	#6 AND #1<br>*Indexes=SCI-EXPANDED, SSCI, A&HCI, CPCI-S, CPCI-SSH, BKCI-S, BKCI-SSH, ESCI, CCR-EXPANDED, IC Timespan=All years*	Edit
# 6	20,138	(TS=((advance* OR future OR prospect* OR forthcoming) NEAR/3 (care OR treatment OR therapy) NEAR/3 (plan* OR intention* OR scheme* OR schedule* OR arrangement* OR measure* OR step* OR intervention* OR procedure* OR action*))) OR (TS=((advance* OR future OR prospect* OR forthcoming) NEAR/3 (directive* OR instruction* OR preferences))) OR (TS=(living NEAR/1 will*))<br>*Indexes=SCI-EXPANDED, SSCI, A&HCI, CPCI-S, CPCI-SSH, BKCI-S, BKCI-SSH, ESCI, CCR-EXPANDED, IC Timespan=All years*	Edit
# 5	808	#4 AND #1<br>*Indexes=SCI-EXPANDED, SSCI, A&HCI, CPCI-S, CPCI-SSH, BKCI-S, BKCI-SSH, ESCI, CCR-EXPANDED, IC Timespan=All years*	Edit
# 4	317,221	#3 OR #2<br>*Indexes=SCI-EXPANDED, SSCI, A&HCI, CPCI-S, CPCI-SSH, BKCI-S, BKCI-SSH, ESCI, CCR-EXPANDED, IC Timespan=All years*	Edit
# 3	64,798	(TS=(decision OR choice)) AND (TS=((doctor OR physician OR professional* OR surgeon) NEAR/3 (patient OR consumer OR family) NEAR/3 (relation* OR communication OR interview OR discussion OR consultation)) OR TS=((interpersonal OR personal OR human) NEAR/3 (relation* OR communicat*)) OR TS=((health OR illness) NEAR/3 (attitude* OR knowledge OR education OR information OR literacy OR behavior OR behaviour)) OR TS=((choice OR cooperative OR co-operative) NEAR/3 (behavior OR behaviour)) OR TS=((patient OR consumer) NEAR/3 (attitude* OR participation* OR refusal OR adherence OR compliance OR nonadherence OR non-adherence OR education OR acceptance OR cooperation OR co-operation OR collaboration* OR information OR misinformation OR education OR disput* OR involv*)))<br>*Indexes=SCI-EXPANDED, SSCI, A&HCI, CPCI-S, CPCI-SSH, BKCI-S, BKCI-SSH, ESCI, CCR-EXPANDED, IC Timespan=All years*	Edit

# 2	275,069	TS=((shar* OR joint OR together OR consensual OR collaborativ* OR cooperativ* OR mutual* OR united OR combined OR conjoint* OR accept* OR informed) NEAR/3 decision*) OR TS=((patient* OR consumer* OR famil* OR personal) NEAR/3 (decision* OR choice OR judg* OR consent* OR consens* OR assent* OR dissent* OR preference* OR agreement OR disagreement OR preference* OR involv*))<br>*Indexes=SCI-EXPANDED, SSCI, A&HCI, CPCI-S, CPCI-SSH, BKCI-S, BKCI-SSH, ESCI, CCR-EXPANDED, IC Timespan=All years*	Edit
# 1	29,024	(TS=((heart or cardiac OR aort*) NEAR/3 (stenos*)) OR TS=(valv* NEAR/3 (disease* OR dysfunction* OR failure OR replac* OR prosthes*)) OR TS=(TAVR OR transcatheter OR transfemoral OR transapical OR transaxillary OR SAVR OR "surgical AVR" OR TAVI)) AND (TS=(aged OR older OR elder* OR geriatric*))<br>*Indexes=SCI-EXPANDED, SSCI, A&HCI, CPCI-S, CPCI-SSH, BKCI-S, BKCI-SSH, ESCI, CCR-EXPANDED, IC Timespan=All years*	Edit

Al

Cc

University of Zurich

Clarivate Analytics

Have hundreds of references to organize and format? Begin to save time with EndNote 20

Clarivate
Accelerating innovation

© 2021 Clarivate Copyright notice Terms of use Privacy statement Cookie policy

Sign up for the Web of Science newsletter Follow us
