# Supplementary material for: Shared decision making and advance care planning: a systematic literature review and novel decision-making model
Source: BMC Med Ethics. 2023 Aug 14;24:64. doi: 10.1186/s12910-023-00944-7 (PMC10426137; doi:10.1186/s12910-023-00944-7)
Supplement: Supplementary file 2 — Additional File 2: Summary of included studies [file 12910_2023_944_MOESM2_ESM.pdf]

Table 1. Summary of included studies

Author, Year	Population	Age range	Country	Number included	Males	Study type	Data collection
Quantitative studies							
Korteland, 2017	Patients accepted for aortic and mitral valve replacement	22-84	Netherlands	155 (78 control; 77 intervention)	104	Multicenter prospective randomised controlled trial	Questionnaires delivered pre- and post-surgery
Anaya, 2019	Patients with AS or regurgitation considered for SAVR	45-74	USA	23	17	Non-randomised intervention	Questionnaires
Coylewright, 2020	Patients with symptomatic AS	Mean (SD) = 85.8 (7.8)	USA	35	15	Non-randomised pre-post intervention	Questionnaires
Schmied, 2015	Patients 2 years after aortic valve replacement or reconstruction	66.9 ± 14.2	Germany	468	328	Retrospective survey	Questionnaires
Korteland, 2015	Patients accepted for elective aortic valve replacement	23-86	Netherlands	132	89	Pre-post study	Questionnaires
Sugiura, 2022	Patients with symptomatic severe AS who underwent TAVR	82-89	Japan	98	25	Pre-procedural cross-sectional survey	Questionnaires
Bryssinck, 2021	Patient operated for aortic valve replacement	62-68	Belgium	113	83	Post hock survey	Questionnaires
Dharmarajan, 2017	Patients with sever AS who underwent TAVI, SAVR or medical treatment	34-61	USA	407	237	Retrospective survey	Questionnaires
Qualitative studies							
Skaar, 2017	Elderly patients that recently underwent TAVI	73-89	Norway	10	4	Qualitative study	Semi-structured individual interviews
Coylewright, 2016	Elderly patients eligible for TAVI and their relatives	68-100	USA	46	25	Qualitative retrospective study	Retrospective, qualitative review of the documented patient-defined goals
Olsson, 2016	Patients with severe AS planned for a TAVI treatment	Mean (SD) = 80.7 (7.4)	Sweden	24	15	Qualitative study	Interviews
Beishuizen, 2021	Patients who underwent elective TAVI	Mean (SD) = 81.5 (5.8)	Netherlands	74	28	Observational prospective cohort study	Post operative surveying and interviews
Ingle, 2021	Patients that underwent TAVR or SAVR	48-92	USA	17	10	Qualitative study	In-depth, semi-structured interviews
Picou, 2022	3 cohorts of patients with AS: with previous TAVI, with previous SAVR, current medication	≥40	USA	45	25	Qualitative study	Semi-structured interviews
Col, 2022	Patients with severe AS who underwent decision-making regarding AS treatment	36-92	USA	51	28	Multi-center mixed methods qualitative study	Nominal group technique meetings (focus groups)

AS – aortic stenosis; TAVI – transcatheter aortic valve implantation; SAVR - surgical aortic valve replacement; SD – standard deviation

Inclusion criteria: The literature search was conducted in CINAHL, Cochrane, EMBASE, MEDLINE, PsychINFO, Scopus and Web of Science (see supplementary file). The search strategy included all empirical studies (randomised, quasi randomised trials and qualitative studies) focusing on patients with symptomatic AS, their surrogates and/or their healthcare professionals (HCP). We searched for studies that described SDM and/or ACP or other communication interventions used to aid the decision-making process between TAVI vs. SAVR, TAVI vs. no intervention and SAVR vs. no intervention. The intervention was defined as any form of communication related to patient's needs, perspectives, beliefs, feelings, understanding, experiences, values and preferences concerning AS treatment options for immediate care, or in case of decision-making incapacity as a results of treatment complication. Outcomes were defined as assessment of the decisionmaking process, of quality of communication, informed choice, patient reported quality of life (also disease-related), health status reported by patients, surrogates and their HCP, adherence to goals of care, decisional conflict. Papers in languages other than German or English, case reports, reviews or meta-analyses were excluded from this review. Papers that report the decision-making process from perspectives other than those of the patient were also excluded (ex. decisions made by heart teams alone). The setting included general practitioners' offices, cardiologic outpatient clinics, and inpatient clinics.
